# Inhibition of Xanthine Oxidase-Catalyzed Xanthine and 6-Mercaptopurine Oxidation by Flavonoid Aglycones and Some of Their Conjugates

**DOI:** 10.3390/ijms21093256

**Published:** 2020-05-05

**Authors:** Violetta Mohos, Eszter Fliszár-Nyúl, Miklós Poór

**Affiliations:** 1 Department of Pharmacology, Faculty of Pharmacy, University of Pécs, Szigeti út 12, H-7624 Pécs, Hungary; mohos.violetta@gytk.pte.hu (V.M.); eszter.nyul@aok.pte.hu (E.F.-N.); 2 János Szentágothai Research Centre, University of Pécs, Ifjúság útja 20, H-7624 Pécs, Hungary

**Keywords:** flavonoids, flavonoid conjugates, xanthine oxidase, xanthine, 6-mercaptopurine, biotransformation, food-drug interaction

## Abstract

Flavonoids are natural phenolic compounds, which are the active ingredients in several dietary supplements. It is well-known that some flavonoid aglycones are potent inhibitors of the xanthine oxidase (XO)-catalyzed uric acid formation *in vitro*. However, the effects of conjugated flavonoid metabolites are poorly characterized. Furthermore, the inhibition of XO-catalyzed 6-mercaptopurine oxidation is an important reaction in the pharmacokinetics of this antitumor drug. The inhibitory effects of some compounds on xanthine vs. 6-mercaptopurine oxidation showed large differences. Nevertheless, we have only limited information regarding the impact of flavonoids on 6-mercaptopurine oxidation. In this study, we examined the interactions of flavonoid aglycones and some of their conjugates with XO-catalyzed xanthine and 6-mercaptopurine oxidation *in vitro*. Diosmetin was the strongest inhibitor of uric acid formation, while apigenin showed the highest effect on 6-thiouric acid production. Kaempferol, fisetin, geraldol, luteolin, diosmetin, and chrysoeriol proved to be similarly strong inhibitors of xanthine and 6-mercaptopurine oxidation. While apigenin, chrysin, and chrysin-7-sulfate were more potent inhibitors of 6-mercaptopurine than xanthine oxidation. Many flavonoids showed similar or stronger (even 5- to 40-fold) inhibition of XO than the positive control allopurinol. Based on these observations, the extremely high intake of flavonoids may interfere with the elimination of 6-mercaptopurine.

## 1. Introduction

Flavonoids are plant-derived phenolic compounds found in numerous fruits, vegetables, and spices [1]. Several studies suggest the beneficial health effects of flavonoids (e.g., antioxidant, anti-inflammatory, and antiproliferative actions), therefore, many dietary supplements contain high doses of flavonoids (even more ten- or hundred-fold vs. the normal dietary intake) [2,3]. Typically, flavonoids have low oral bioavailability, due to their significant metabolism in enterocytes and hepatocytes [4]. The biotransformation of flavonoids by catechol-*O*-methyltransferase (COMT), sulfotransferase (SULT), and uridine diphosphate glucuronosyltransferase (UGT) results in the formation of methyl, sulfate, and glucuronide conjugates, respectively [5]. These derivatives commonly reach considerably higher concentrations in the systemic circulation than the parent compound [6,7]. Flavonoid chrysin (CHR) is extensively biotransformed by SULT and UGT, its dominant metabolites are chrysin-7-sulfate (C7S) and chrysin-7-glucuronide (C7G) in the human circulation [8]. COMT is also a frequently involved enzyme in flavonoid metabolism, the 3’-*O*-methylation of fisetin (FIS) results in geraldol (3’-*O*-methylfisetin, GER) [9,10], while diosmetin (4’-*O*-methylluteolin, DIO) and chrysoeriol (3’-*O*-methylluteolin, CHE) are formed from luteolin (LUT) by COMT [11]. LUT is a rare substrate of COMT because its 4’-*O*-substitution is preferred (vs. the typical 3’-*O*-methylation) [12]. Furthermore, DIO is the dominant circulating metabolite of orally-administered diosmin, the latter is the active ingredient of several medications (e.g., Detralex^®^ and Daflon^®^) and dietary supplements [13]. Methylated flavonoids are not only produced during the biotransformation in mammals, but also found in certain plants [14,15,16]. GER appears in *Trifolium subterraneum* [16,17], while CHE is a constituent of rooibos tea (*Aspalathus linearis*) and of *Digitalis purpurea* [14,15].

**Figure 1:**
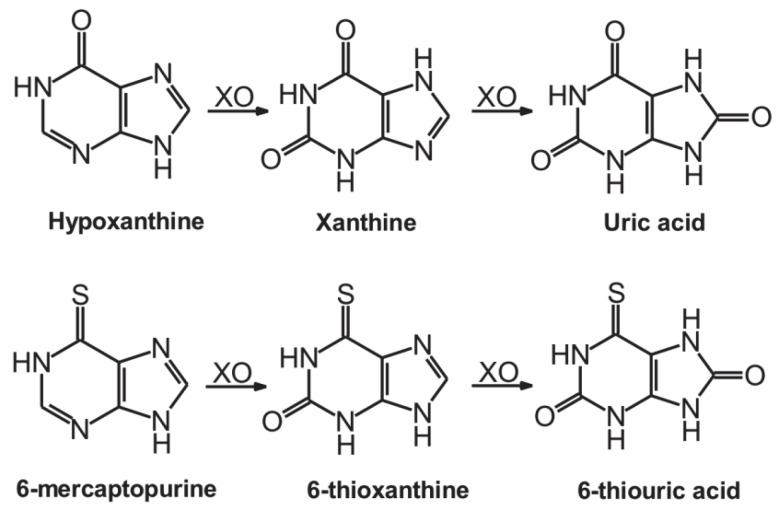
XO-catalyzed oxidation of hypoxanthine to uric acid (**top**) and 6-mercaptopurine to 6-thiouric acid (**bottom**).

Xanthine oxidase (XO) is a non-microsomal molybdenum-containing enzyme involved in purine catabolism. XO oxidizes hypoxanthine to xanthine then to uric acid (Figure 1) [18]. Allopurinol is a potent inhibitor of XO, it is used as a medication to treat hyperuricemia or gout [18]. Allopurinol competitively inhibits XO and it is oxidized to oxipurinol, which in turn is a potent pseudo-irreversible inhibitor of XO [19]. Inhibition of XO results in the decreased formation of uric acid and also reduces the XO-mediated formation of superoxide anion radicals in some pathological conditions [20]. Furthermore, XO is also involved in the biotransformation of the antitumor drug 6-mercaptopurine (6MP) (Figure 1) [21]. Considering the fact that the simultaneous administration of allopurinol and 6MP slows down the elimination of the latter compound, the strong inhibition of XO-catalyzed 6MP oxidation can cause even serious myelosuppression [22].

The strong inhibition of XO by flavonoids has been reported in several *in vitro* studies [23,24,25,26,27]. Based on previous investigations, flavonoid aglycones apigenin (API), CHR, FIS, kaempferol (KAE), and LUT are potent inhibitors of the XO-catalyzed xanthine oxidation [23,26]. Besides their strong effects on XO, we selected these aglycones because they are contained in many dietary supplements widely marketed through the Internet. The extremely high intake of flavonoids (as a result of the consumption of dietary supplements) can lead to the high plasma concentrations of flavonoids and/or their metabolites [6,7,13]. Xanthine is the generally applied substrate in XO assays; however, the inhibitory effects of flavonoids on XO-catalyzed 6MP oxidation has been poorly studied. In our recent report, we demonstrated that quercetin as well as its sulfate and methyl conjugates are similarly potent inhibitors of xanthine and 6MP oxidation, while some inhibitors (e.g., 3-phenylpropionic acid, pyrogallol, and allopurinol) showed considerably stronger effects on xanthine or on 6MP oxidation [19].

**Figure 2:**
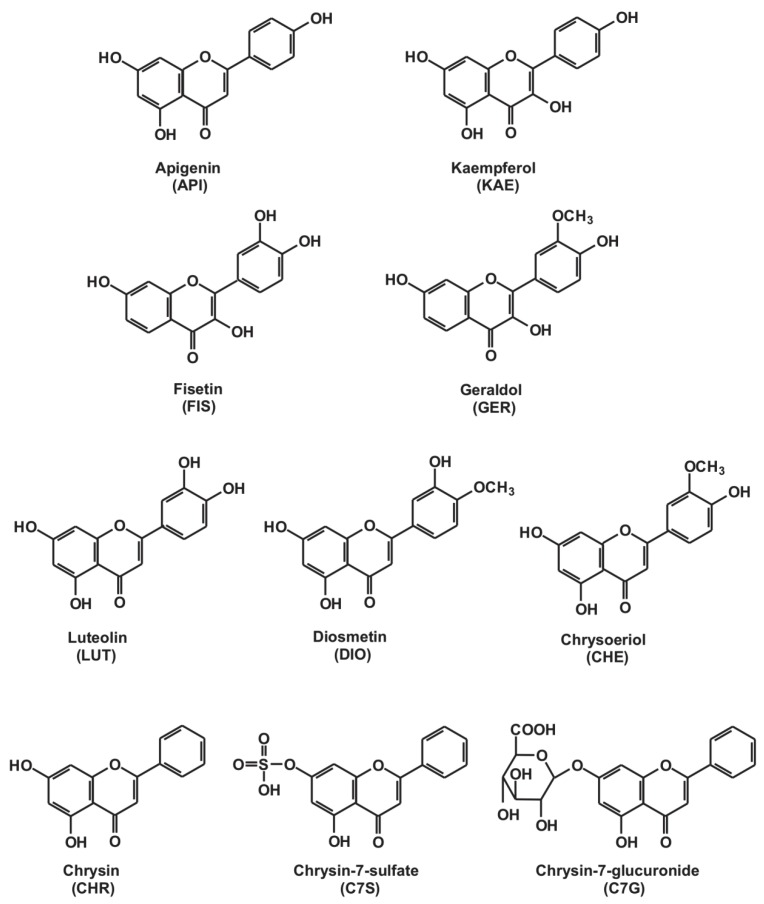
Chemical structures of apigenin (5,7,4’-trihydroxyflavone), kaempferol (3,5,7,4′-tetrahydroxyflavone), fisetin (3,7,3′,4′-tetrahydroxyflavone), geraldol (3’-*O*-methylfisetin), luteolin (5,7,3′,4′-tetrahydroxyflavone), diosmetin (4’-*O*-methylluteolin), chrysoeriol (3’-*O*-methylluteolin), chrysin (5,7-dihydroxyflavone), chrysin-7-sulfate, and chrysin-7-glucuronide.

The two main goals of this study were the following: (1) Comparison of the inhibitory potency of flavonoid conjugates vs. the parent compounds; (2) comparison of the effects of flavonoids on XO-catalyzed xanthine vs. 6MP oxidation. Therefore, the influence of the above-listed flavonoid aglycones and their metabolites (Figure 2) on the XO enzyme were tested employing *in vitro* enzyme assays (using allopurinol as positive control). After incubations, the substrates (xanthine and 6MP) and metabolites (uric acid and 6-thiouric acid) were quantified with high-performance liquid chromatography (HPLC).

## 2. Results

### 2.1. Inhibition of Xanthine and 6-Mercaptopurine Oxidation by Flavonoid Aglycones

**Figure 3:**
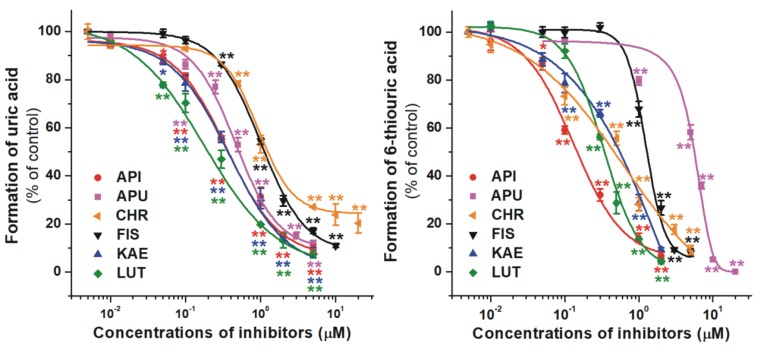
Concentration-dependent inhibitory effects of flavonoid aglycones and the positive control allopurinol (APU) on the XO enzyme. Inhibition of XO-catalyzed uric acid (**left**) and 6-thiouric acid (**right**) formation in the presence of increasing inhibitor concentrations (0–20 μM; substrate concentrations: 5 μM in both assays; API, apigenin; CHR, chrysin; FIS, fisetin; KAE, kaempferol; LUT, luteolin; * *p* < 0.05, ** *p* < 0.01).

First, the effects of flavonoid aglycones (API, CHR, FIS, KAE, and LUT) on the XO-catalyzed xanthine and 6MP oxidation were examined. Each flavonoid tested was able to inhibit both uric acid and 6-thiouric acid formation at nanomolar or low micromolar concentrations (Figure 3). Regarding xanthine oxidation, LUT was the strongest while CHR was the weakest inhibitor (Table 1); and each aglycone showed similar inhibitory potency to allopurinol. However, API and FIS were the strongest and weakest inhibitors of 6MP oxidation, respectively (Figure 3, right). In addition, each flavonoid aglycone was considerably stronger inhibitor of XO-catalyzed 6-thiouric acid formation compared to allopurinol (Table 1).

### 2.2. Inhibition of Xanthine and 6-Mercaptopurine Oxidation by Flavonoid Conjugates

**Figure 4:**
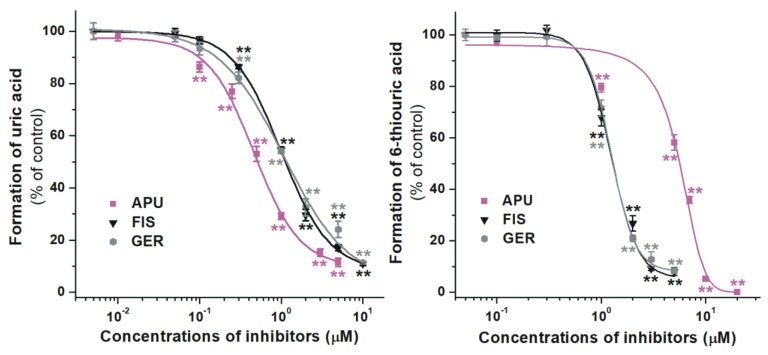
Concentration-dependent inhibitory effects of FIS, GER, and allopurinol (APU) on XO enzyme. Inhibition of XO-catalyzed uric acid (**left**) and 6-thiouric acid (**right**) formation in the presence of increasing inhibitor concentrations (0–20 μM; substrate concentrations: 5 μM in both assays; FIS, fisetin; GER, geraldol; ** *p* < 0.01).

The inhibitory effects of some flavonoid metabolites on XO enzyme were also investigated. Figure 4 demonstrates the formation of uric acid and 6-thiouric acid compared to the control, in the presence of increasing concentrations of FIS and its 3’-*O*-methyl derivative GER. Our results showed that GER induced similarly strong inhibition on both xanthine and 6MP oxidation to FIS (Table 1). Furthermore, FIS and GER were weaker inhibitors of uric acid formation than allopurinol, while they exerted significantly stronger effects on 6-thiouric acid production compared to the positive control (Figure 4, right).

**Figure 5:**
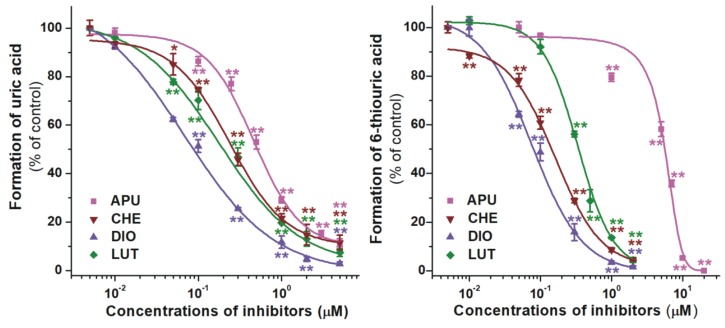
Concentration-dependent inhibitory effects of LUT, DIO, CHE, and allopurinol (APU) on XO enzyme. Inhibition of XO-catalyzed uric acid (**left**) and 6-thiouric acid (**right**) formation in the presence of increasing inhibitor concentrations (0–20 μM; substrate concentrations: 5 μM in both assays; CHE, chrysoeriol; DIO, diosmetin; LUT, luteolin; * *p* < 0.05, ** *p* < 0.01).

The effects of LUT and its methylated metabolites DIO (4’-*O*-methylluteolin) and CHE (3’-*O*-methylluteolin) on XO were also tested. DIO and CHE were stronger and weaker inhibitors of uric acid formation than LUT, respectively (Figure 5, left). Furthermore, both metabolites showed stronger inhibitory effects on uric acid formation compared to allopurinol (Table 1). DIO and CHE proved to be more potent inhibitors of 6MP oxidation vs. the parent compound (Figure 5, right), showing considerably stronger effect than the positive control (Table 1).

**Figure 6:**
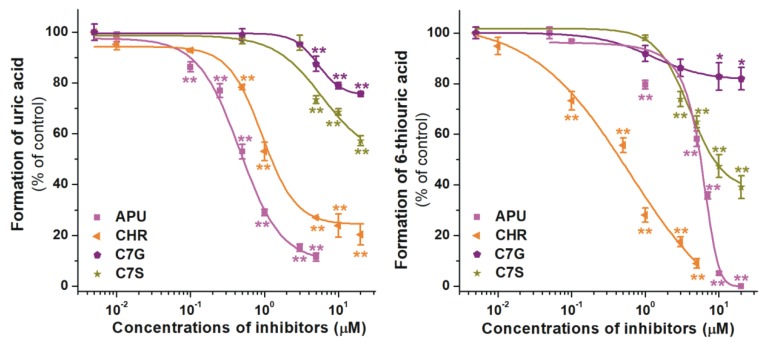
Concentration-dependent inhibitory effects of CHR, C7S, C7G, and allopurinol (APU) on XO enzyme. Inhibition of XO-catalyzed uric acid (**left**) and 6-thiouric acid (**right**) formation in the presence of increasing inhibitor concentrations (0–20 μM; substrate concentrations: 5 μM in both assays; C7G, chrysin-7-glucuronide; C7S, chrysin-7-sulfate; CHR, chrysin; * *p* < 0.05, ** *p* < 0.01).

Finally, the effects of chrysin as well as its sulfate and glucuronide metabolites on XO were examined. Although, C7S and C7G exerted statistically significant inhibition on XO-catalyzed xanthine and 6MP oxidation, they proved to be considerably weaker inhibitors vs. the parent compound in both assays (Figure 6). C7G was a poor inhibitor of XO, while C7S did not induce a 50% decrease in the metabolite formation in the xanthine assay, even at four-fold concentration compared to the substrate. Furthermore, CHR showed weaker and considerably stronger inhibitory effects on xanthine and 6MP oxidation than allopurinol, respectively (Figure 6 and Table 1).

## 3. Discussion

The strong *in vitro* inhibitory effects of API, CHR, FIS, KAE, and LUT on the XO-catalyzed xanthine oxidation have been widely investigated in earlier studies [23,24,25,26,27]. However, the effects of their conjugated metabolites on xanthine oxidation as well as the inhibitory action of these flavonoids on 6MP oxidation has not been or has been only poorly examined. Despite the fact that earlier studies characterized the strong inhibitory potential of flavonoids vs. xanthine oxidation, some studies suggest their similar [26,28], stronger [24,27], or even weaker [23,25,29] effects compared to allopurinol. Regarding the flavonoids examined in our study, their IC_50_ values showed large variances in previous investigations (API: 0.7 to 3.6 μM, CHR: 0.8 to 5.0 μM, FIS: 4.3 to 11.3 μM, KAE: 0.7 to 16.9 μM, LUT: 0.6 to 8.8 μM) [23,24,25,26,27,28,29]. Nevertheless, our results are consistent with previous reports, suggesting the nanomolar or low micromolar IC_50_ values regarding flavonoid aglycones tested in the current study (Table 1). Previously reported data are highly controversial regarding CHE: negligible [30], mild [31], and strong [32] inhibition of XO has been also described. In earlier studies, DIO proved to be a strong inhibitor of xanthine oxidation, however, both its weaker [25,33] and stronger [32] effects vs. allopurinol have been reported. Furthermore, we did not find any data regarding the impacts of GER, C7S, and C7G on XO. Similarly to C7G (Figure 6, right), the pharmacologically relevant glucuronide conjugates of quercetin were poor inhibitors of xanthine oxidation [19]. Interestingly, quercetin-3’-glucuronide and quercetin-4’-glucuronide exerted strong inhibitory effects on xanthine oxidation [34]; however, these conjugates are not typical in the human body. Methyl conjugates tested in this study (CHE, DIO, and GER) were similar or stronger inhibitors of the enzyme than the parent flavonoids (Table 1), which is in agreement with previous observations regarding LUT [32] and quercetin [19]. C7S showed significantly weaker effect than CHR (Table 1); while in another study, quercetin-3’-sulfate and quercetin were equally strong inhibitors of xanthine oxidation [19].

For the appropriate comparison, both xanthine and 6MP assays were performed with 5 μM substrate concentrations. It is important to note that, in the 6MP assay, the considerably stronger inhibitory effects of flavonoids vs. the positive control are resulted from the fact that allopurinol is a more than five-fold stronger inhibitor of xanthine than 6MP oxidation (Table 1). Under the applied conditions, C7G was the sole compound which showed only weak inhibition in both assays. Similar observations have been reported regarding quercetin-3-glucuronide and isorhamnetin-3-glucuronide [19,34]. Some of the flavonoids (CHE, DIO, FIS, GER, KAE, and LUT) showed same inhibitory effects on XO-catalyzed xanthine and 6MP oxidation. However, API, CHR, and C7S were more potent inhibitors of 6-thiouric acid vs. uric acid formation (Table 1), similarly to several colonic flavonoid metabolites (e.g., 3-phenylpropionic acid, 4-methoxysalicylic acid, and 3-coumaric acid) [19]. DIO was highly the strongest inhibitor of xanthine oxidation, while the most potent effects on 6MP oxidation were produced by DIO, API, and CHE (the IC_50_ values of these flavonoids were lower than 100 nM).

We cannot directly extrapolate our *in vitro* data; however, based on the previously reported investigations, we may estimate the *in vivo* importance of flavonoid-XO interactions. Based on earlier studies, the *in vivo* effects of flavonoids on xanthine oxidation are controversial. The orally administered API (175–700 mg/kg) [35], LUT (16–100 mg/kg) and LUT-7-*O*-glucoside (50 and 100 mg/kg) [36] did not affect the serum uric acid levels in hyperuricaemic mice. However, in the latter study, LUT (50 mg/kg) induced a slight but significant decrease in uric acid levels after *i.p.* administration [36]. In another study, after *per os* treatment, API (50 and 100 mg/kg), KAE (50 and 100 mg/kg), and LUT (100 mg/kg) significantly decreased serum uric acid concentrations in hyperuricemic mice, and KAE also decreased uric acid levels in normal mice [37]. Only limited clinical data are available, however, the human studies with quercetin suggest its negligible antihyperuricemic effects [38,39,40]. Even the extremely high intake of flavonoids (more hundreds of milligrams to few grams) can lead to approximately ten-fold lower total peak plasma concentrations (flavonoids and their conjugated metabolites jointly) vs. allopurinol and oxipurinol together [13,41,42,43]. Because the inhibitory action of flavonoids on xanthine oxidation is similar to allopurinol (Table 1), it seems to be unlikely that the considerably lower levels of flavonoids can induce similar antihyperuricemic effects to allopurinol. However, some of the flavonoids tested (API, CHE, CHR, DIO, KAE, and LUT) exerted 5- to 40-fold stronger inhibitory effect on XO-catalyzed 6MP oxidation than allopurinol (Table 1), suggesting that even the significantly lower levels of flavonoids (vs. allopurinol/oxipurinol) may affect the elimination of 6MP. Because the simultaneous administration of the conventional therapeutic doses of 6MP and allopurinol can cause serious myelosuppression [22], the potential *in vivo* interaction of flavonoids with 6MP may have high pharmacological/toxicological importance.

Considering the above-listed observations, the possible extremely high intake of flavonoids (e.g., through the consumption of dietary supplements containing several hundreds of milligrams or even more grams of pure aglycones [3,44]) may interfere with the elimination of 6MP. Therefore, it is reasonable to perform *in vivo* studies in the near future to accept or reject this hypothesis.

## 4. Materials and Methods

### 4.1. Reagents

Xanthine oxidase (XO; from bovine milk), xanthine, uric acid, 6-mercaptopurine (6MP), allopurinol, apigenin (API), chrysin (CHR), diosmetin (DIO), fisetin (FIS), and kaempferol (KAE) were purchased from Sigma-Aldrich (St. Louis, MO, United States). Luteolin (LUT), geraldol (GER), and chrysoeriol (CHE) were obtained from Extrasynthese (Genay Cedex, France). Chrysin-7-glucuronide (C7G) and 6-thiouric acid were purchased from Carbosynth (Berkshire, UK). Chrysin-7-sulfate (C7S) was synthetized as described [44,45]. Stock solutions of flavonoids (each 2 mM) were prepared in dimethyl sulfoxide (DMSO) and stored at −20 °C.

### 4.2. XO Assays

To test the inhibitory effects of flavonoids and their metabolites on the XO-catalyzed oxidation of xanthine and 6MP, the previously reported methods were applied without modifications [19]. In both assays, allopurinol was applied as positive control. In each experiment, solvent controls (DMSO) were used. Metabolite formation (% of control) was plotted vs. the inhibitor concentrations in decimal logarithmic scale. IC_50_ values were evaluated by sigmoidal fitting, employing GraphPad Prism 8 software (San Diego, CA, USA).

### 4.3. HPLC Analyses

After the *in vitro* XO assays, xanthine and uric acid as well as 6MP and 6-thiouric acid were quantified by the HPLC-UV methods described in our previous report, without any modifications [19].

### 4.4. Statistics

Figures and table represent means and standard error of the mean (SEM) values (at least from three independent experiments). Statistical differences were analyzed (*p* < 0.05 and *p* < 0.01) employing one-way ANOVA, using Tukey’s post-hoc test (IBM SPSS Statistics; Armonk, NY, USA).

## Figures and Tables

**Table 1:** Inhibitory effects of flavonoids and their conjugated metabolites on XO-catalyzed xanthine and 6MP oxidation.

Test Compound	IC_50(xanthine)_ (μM)	IC_50(6MP)_ (μM)	IC_50 (xanthine)_/IC_50 (6MP)_
Allopurinol	0.38	1.97	0.19
Apigenin	0.27	0.09	3.00
Kaempferol	0.29	0.38	0.76
Fisetin	0.86	1.10	0.78
Geraldol	0.68	1.19	0.57
Luteolin	0.16	0.27	0.59
Diosmetin	0.04	0.05	0.80
Chrysoeriol	0.17	0.09	1.88
Chrysin	0.70	0.22	3.18
Chrysin-7-sulfate	> 20.0	2.23	-
Chrysin-7-glucuronide	> 20.0	> 20.0	-

## Abbreviations

6MP	6-mercaptopurine
API	Apigenin
APU	Allopurinol
C7G	Chrysin-7-glucuronide
C7S	Chrysin-7-sulfate
CHE	Chrysoeriol
CHR	Chrysin
COMT	Catechol-*O*-methyltransferase
DIO	Diosmetin
DMSO	Dimethyl sulfoxide
FIS	Fisetin
GER	Geraldol
HPLC	High-performance liquid chromatography
KAE	Kaempferol
LUT	Luteolin
SEM	Standard error of the mean
SULT	Sulfotransferase
UGT	Uridine diphosphate glucuronosyltransferase
XO	Xanthine oxidase

## References

[1] Cook N.C., Samman S. (1996). Flavonoids—Chemistry, metabolism, cardioprotective effects, and dietary sources. J. Nutr. Biochem..

[2] Nijveldt R.J., Van Nood E., Van Hoorn D.E.C., Boelens P.G., Van Norren K., Van Leeuwen P.A.M. (2001). Flavonoids: A review of probable mechanisms of action and potential applications. Am. J. Clin. Nutr..

[3] Vida R.G., Fittler A., Somogyi-Végh A., Poór M. (2019). Dietary quercetin supplements: Assessment of online product informations and quantitation of quercetin in the products by high-performance liquid chromatography. Phytother. Res..

[4] Manach C., Donovan J.L. (2004). Pharmacokinetics and Metabolism of Dietary Flavonoids in Humans. Free Radic. Res..

[5] Chen Z., Zheng S., Li L., Jiang H. (2014). Metabolism of Flavonoids in Human: A Comprehensive Review. Curr. Drug Metab..

[6] Wang L., Morris M.E. (2005). Liquid chromatography–tandem mass spectroscopy assay for quercetin and conjugated quercetin metabolites in human plasma and urine. J. Chromatogr..

[7] Mullen W., Edwards C.A., Crozier A. (2006). Absorption, Excretion and Metabolite Profiling of Methyl-, Glucuronyl-, Glucosyl- and Sulpho-Conjugates of Quercetin in Human Plasma and Urine After Ingestion of Onions. Br. J. Nutr..

[8] Walle T., Otake Y., Brubaker J.A., Walle U.K., Halushka P.V. (2001). Disposition and metabolism of the flavonoid chrysin in normal volunteers. Br. J. Clin. Pharmacol..

[9] Khan N., Syed D.N., Ahmad N., Mukhtar H. (2013). Fisetin: A Dietary Antioxidant for Health Promotion. Antioxid. Redox Signal..

[10] Touil Y.S., Auzeil N., Boulinguez F., Saighi H., Regazzetti A., Scherman D., Chabot G.G. (2011). Fisetin disposition and metabolism in mice: Identification of geraldol as an active metabolite. Biochem. Pharmacol..

[11] Chen Z., Chen M., Pan H., Sun S., Li L., Zeng S., Jiang H. (2011). Role of Catechol-O-Methyltransferase in the Disposition of Luteolin in Rats. Drug Metab. Dispos..

[12] Chen Z.J., Dai Y.Q., Kong S.S., Song F.F., Li L.P., Ye J.F., Wang R.W., Zeng S., Zhou H., Jiang H.D. (2013). Luteolin is a rare substrate of human catechol-O-methyltransferase favoring a para-methylation. Mol. Nutr. Food Res..

[13] Campanero M.A., Escolar M., Perez G., Garcia-Quetglas E., Sadaba B., Azanza J.R. (2010). Simultaneous determination of diosmin and diosmetin in human plasma by ion trap liquid chromatography–atmospheric pressure chemical ionization tandem mass spectrometry: Application to a clinical pharmacokinetic study. J. Pharmac. Biomed. Anal..

[14] Khan A.U., Gilani A.H. (2006). Selective bronchodilatory effect of Rooibos tea (Aspalathus linearis) and its flavonoid, chrysoeriol. Eur. J. Nutr..

[15] Choi D.Y., Lee J.Y., Kim M.R., Woo E.R., Kim Y.G., Kang K.W. (2005). Chrysoeriol potently inhibits the induction of nitric oxide synthase by blocking AP-1 activation. J. Biomed. Sci..

[16] Wong E., Francis C.M. (1968). Flavonoids in genotypes of Trifolium subterraneum—I: The normal flavonoid pattern of the Geraldton variety. Phytochemistry.

[17] Gupta S.R., Ravindranath B., Seshadri T.R. (1971). Synthesis of some flavonoid glucosides of trifolium subterraneum. Phytochemistry.

[18] Day R.O., Graham G.G., Hicks M., McLachlan A.J., Stocker S.L., Williams K.M. (2007). Clinical Pharmacokinetics and Pharmacodynamics of Allopurinol and Oxypurinol. Clin. Pharmacokinet..

[19] Mohos V., Pánovics A., Fliszár-Nyúl E., Schilli G., Hetényi C., Mladěnka P., Needs P.W., Kroon P.A., Pethő G., Poór M. (2019). Inhibitory Effects of Quercetin and Its Human and Microbial Metabolites on Xanthine Oxidase Enzyme. Int. J. Mol. Sci..

[20] Galbusera C., Orth P., Fedida D., Spector T. (2006). Superoxide radical production by allopurinol and xanthine oxidase. Biochem. Pharmacol..

[21] Leong R.W., Gearry R.B., Sparrow M.P. (2008). Thiopurine hepatotoxicity in inflammatory bowel disease: The role for adding allopurinol. Expert Opin. Drug Saf..

[22] McLeod H.L. (1998). Clinically relevant drug–drug interactions in oncology. Br. J. Clin. Pharmacol..

[23] Nagao A., Seki M., Kobayashi H. (1999). Inhibition of Xanthine Oxidase by Flavonoids. Biosci. Biotechnol. Biochem..

[24] Lin S., Zhang G., Liao Y., Pan J. (2015). Inhibition of chrysin on xanthine oxidase activity and its inhibition mechanism. Int. J. Biol. Macromol..

[25] Lin S., Zhang G., Liao Y., Pan J., Gong D. (2015). Dietary Flavonoids as Xanthine Oxidase Inhibitors: Structure–Affinity and Structure–Activity Relationships. J. Agric. Food Chem..

[26] Cos P., Ying L., Calomme M., Hu J.P., Cimanga K., Van Poel B., Pieters L., Vlietinck A.J., Vanden Berghe D. (1998). Structure−Activity Relationship and Classification of Flavonoids as Inhibitors of Xanthine Oxidase and Superoxide Scavengers. J. Nat. Prod..

[27] Van Hoorn D.E.C., Nijveldt R.J., Van Leeuwen P.A.M., Hofman Z., M’Rabet L., De Bont D.B.A., Van Norren K. (2002). Accurate prediction of xanthine oxidase inhibition based on the structure of flavonoids. Eur. J. Pharmacol..

[28] Lin C.M., Chen C.S., Chen C.T., Liang Y.C., Lin J.K. (2002). Molecular modeling of flavonoids that inhibits xanthine oxidase. Biochem. Biophys. Res. Commun..

[29] Iio M., Moriyama A., Matsumoto Y., Takaki N., Fukumoto M. (1985). Inhibition of Xanthine Oxidase by Flavonoids. Agric. Biol. Chem..

[30] Cimanga K., De Bruyne T., Hu J.P., Cos P., Apers S., Pieters L., Tona L., Kambu K., Vanden Berghe D., Vlietinck A.J. (1999). Constituents from Morinda morindoides Leaves as Inhibitors of Xanthine Oxidase and Scavengers of Superoxide Anions. Pharm. Pharmacol. Commun..

[31] Hayashi T., Sawa K., Kawasaki M., Arisawa M., Shimizu M., Morita N. (1988). Inhibition of cow’s milk xanthine oxidase by flavonoids. J. Nat. Prod..

[32] Nguyen M.T.T., Awale S., Tezuka Y., Ueda J., Tran Q.L., Kadota S. (2006). Xanthine Oxidase Inhibitors from the Flowers of Chrysanthemum sinense. Planta Med..

[33] Qu L., Ruan J.Y., Jin L.J., Shi W.Z., Li X.X., Han L.F., Zhang Y., Wang T. (2017). Xanthine oxidase inhibitory effects of the constituents of Chrysanthemum morifolium stems. Phytochem. Lett..

[34] Day A.J., Bao Y., Morgan M.R., Williamson G. (2000). Conjugation position of quercetin glucuronides and effect on biological activity. Free Radic. Biol. Med..

[35] Huang J., Wang S., Zhu M., Chen J., Zhu X., Chen J., Zhu X. (2011). Effects of Genistein, Apigenin, Quercetin, Rutin and Astilbin on serum uric acid levels and xanthine oxidase activities in normal and hyperuricemic mice. Food Chem. Toxicol..

[36] Sarawek S., Feistel B., Pischel I., Butterweck V. (2008). Flavonoids of Cynara scolymus Possess Potent Xanthinoxidase Inhibitory Activity in vitro but are Devoid of Hypouricemic Effects in Rats after Oral Application. Planta Med..

[37] Mo S.F., Zhou F., Lv Y.Z., Hu Q.H., Zhang D.M., Kong L.D. (2007). Hypouricemic Action of Selected Flavonoids in Mice: Structure–Activity Relationships. Biol. Pharm. Bull..

[38] Abbey E.L., Rankin J.W. (2011). Effect of quercetin supplementation on repeated-sprint performance, xanthine oxidase activity, and inflammation. Int. J. Sport Nutr. Exerc. Metab..

[39] Boots A.W., Drent M., De Boer V.C., Bast A., Haenen G.R. (2011). Quercetin reduces markers of oxidative stress and inflammation in sarcoidosis. Clin. Nutr..

[40] Shi Y., Williamson G. (2016). Quercetin lowers plasma uric acid in pre-hyperuricaemic males: A randomised, double-blinded, placebo-controlled, cross-over trial. Br. J. Nutr..

[41] Cao J., Zhang Y., Chen W., Zhao X. (2010). The relationship between fasting plasma concentrations of selected flavonoids and their ordinary dietary intake. Br. J. Nutr..

[42] Conquer J.A., Maiani G., Azzini E., Raguzzini A., Holub B.J. (1998). Supplementation with Quercetin Markedly Increases Plasma Quercetin Concentration without Effect on Selected Risk Factors for Heart Disease in Healthy Subjects. J. Nutr..

[43] Turnheim K., Krivanek P., Oberbauer R. (1999). Pharmacokinetics and pharmacodynamics of allopurinol in elderly and young subjects. Br. J. Clin. Pharmacol..

[44] Mohos V., Fliszár-Nyúl E., Schilli G., Hetényi C., Lemli B., Kunsági-Máté S., Bognár B., Poór M. (2018). Interaction of Chrysin and Its Main Conjugated Metabolites Chrysin-7-Sulfate and Chrysin-7-Glucuronide with Serum Albumin. Int. J. Mol. Sci..

[45] Huang W.H., Lee A.R., Yang C.H. (2006). Antioxidative and anti-inflammatory activities of polyhydroxyflavonoids of Scutellaria baicalensis GEORGI. Biosci. Biotechnol. Biochem..

